# Cooperative Micromanipulation Using the Independent Actuation of Fifty Microrobots in Parallel

**DOI:** 10.1038/s41598-017-03525-y

**Published:** 2017-06-12

**Authors:** M. Arifur Rahman, Julian Cheng, Zhidong Wang, Aaron T. Ohta

**Affiliations:** 1 Dept. of Electrical Engineering, University of Hawaii at Manoa, Honolulu, Hawaii USA; 2 Dept. of Advanced Robotics, Chiba Institute of Technology, Narashino, Chiba Japan

## Abstract

Micromanipulation for applications in areas such as tissue engineering can require mesoscale structures to be assembled with microscale resolution. One method for achieving such manipulation is the parallel actuation of many microrobots in parallel. However, current microrobot systems lack the independent actuation of many entities in parallel. Here, the independent actuation of fifty opto-thermocapillary flow-addressed bubble (OFB) microrobots in parallel is demonstrated. Individual microrobots and groups of microrobots were moved along linear, circular, and arbitrary 2D trajectories. The independent addressing of many microrobots enables higher-throughput microassembly of micro-objects, and cooperative manipulation using multiple microrobots. Demonstrations of manipulation with multiple OFB microrobots include the transportation of microstructures using a pair or team of microrobots, and the cooperative manipulation of multiple micro-objects. The results presented here represent an order of magnitude increase in the number of independently actuated microrobots in parallel as compared to other magnetically or electrostatically actuated microrobots, and a factor of two increase as compared to previous demonstrations of OFB microrobots.

## Introduction

Microrobots are untethered sub-millimeter actuators capable of manipulating micro-objects including living cells^1–9^, and are useful for applications such as targeted therapeutics^1^, molecular delivery to targeted cells^10^, minimally invasive surgery^11^, and tissue engineering^12, 13^. Microrobots can be actuated using a wide variety of methods, including electrostatic actuation^14, 15^, magnetic actuation^2, 4, 6–8, 16–18^, a combination of electrostatic and magnetic actuation^19, 20^, optothermal actuation^3, 5, 21–24^, a combination of acoustic and optothermal actuation^25^, and bacteria-propelled swimming^26–29^. Micro-manipulation using multiple microrobots can increase assembly throughput and reduce manipulation time. For example, a large team of independently controlled microrobots can also split into multiple smaller groups to carry out various micromanipulation tasks in parallel^30^. Moreover, using many microrobots in parallel allows the cooperative manipulation of micro-objects that are too heavy or unwieldy to be transported by a few microrobots. Swarms of multiple microrobots are suitable for the assembly of cell-laden microgels, which are used to create *in vitro* tissue constructs^13, 31^. Similarly, an independently controlled swarm of microrobots can perform molecular or drug delivery^10^ to multiple specific cells simultaneously with single-cell resolution.

Electrostatic and electromagnetic actuation can manipulate multiple microrobots in parallel, but it is challenging to move microrobots along independent trajectories using global actuation signals that couple microrobot motion to one another. One solution is to vary the physical properties of the microrobots so that each one has a different response to a global actuation signal^14, 15, 32^. Another approach is the use of specialized working surfaces with arrays of transducers that create localized actuation forces^19, 32, 33^. This enables the simultaneous actuation of microrobots along different trajectories, but has so far been limited to 10 microrobots or less. The motion of bacteria-propelled microrobots is uncoupled to one another, but current levels of control of the trajectories of these microrobots are less precise than other types of microrobots. To summarize, despite progress in the parallel actuation of microrobots, it remains challenging to actuate many microrobots independently; the highest number to date using electrostatic or electromagnetic actuation is eight microrobots^14^.

A microrobot actuation mechanism that allows the parallel actuation of many microrobots is opto-thermal actuation, in which optical energy is converted to thermal energy. This is the mechanism utilized by opto-thermocapillary flow-addressed bubble (OFB) microrobots, which are gas bubbles in liquid media that move along optically generated thermal gradients^22^. OFB microrobots are capable of microassembly^22^, single-cell assembly^3, 5^, cell-laden hydrogel assembly^3^, and single-cell poration^10^. Compared to other methods of actuation discussed above, OFB microrobots have less dependence on the electrical and magnetic properties of both the object under manipulation and the media used for actuation. More importantly, each OFB microrobot is optically addressed, so it is straightforward to maintain independent control even when moving many microrobots at once. For this work, “independent control” means the uncoupled actuation of individual microrobots, allowing motion along different trajectories at different velocities.

Previously, 10 OFB microrobots were moved in parallel, but the movement of each microrobot was not independent^23^. Subsequently, up to 24 OFB microrobots were generated and independently actuated on a glass substrate coated with amorphous silicon^24^. This paper presents the independent actuation of 50 microrobots in parallel on a titanium-coated glass slide. The microrobots were simultaneously maneuvered in different directions to demonstrate uncoupled parallel actuation. The multidirectional maneuverability of the microrobots along various trajectories was demonstrated, and provided the flexibility needed to manipulate micro-objects into various orientations. The independent actuation of multiple OFB microrobots enables cooperative micromanipulation, and enhances the assembly capabilities of this microrobot system. Multiple OFB microrobots working together are capable of exerting more force on microstructures. In addition, cooperative micromanipulation by a team of OFB microrobots was used to simultaneously transport multiple micro-objects in less time compared to manipulation using a single microrobot.

### OFB microrobot generation and actuation

**Figure 1:**
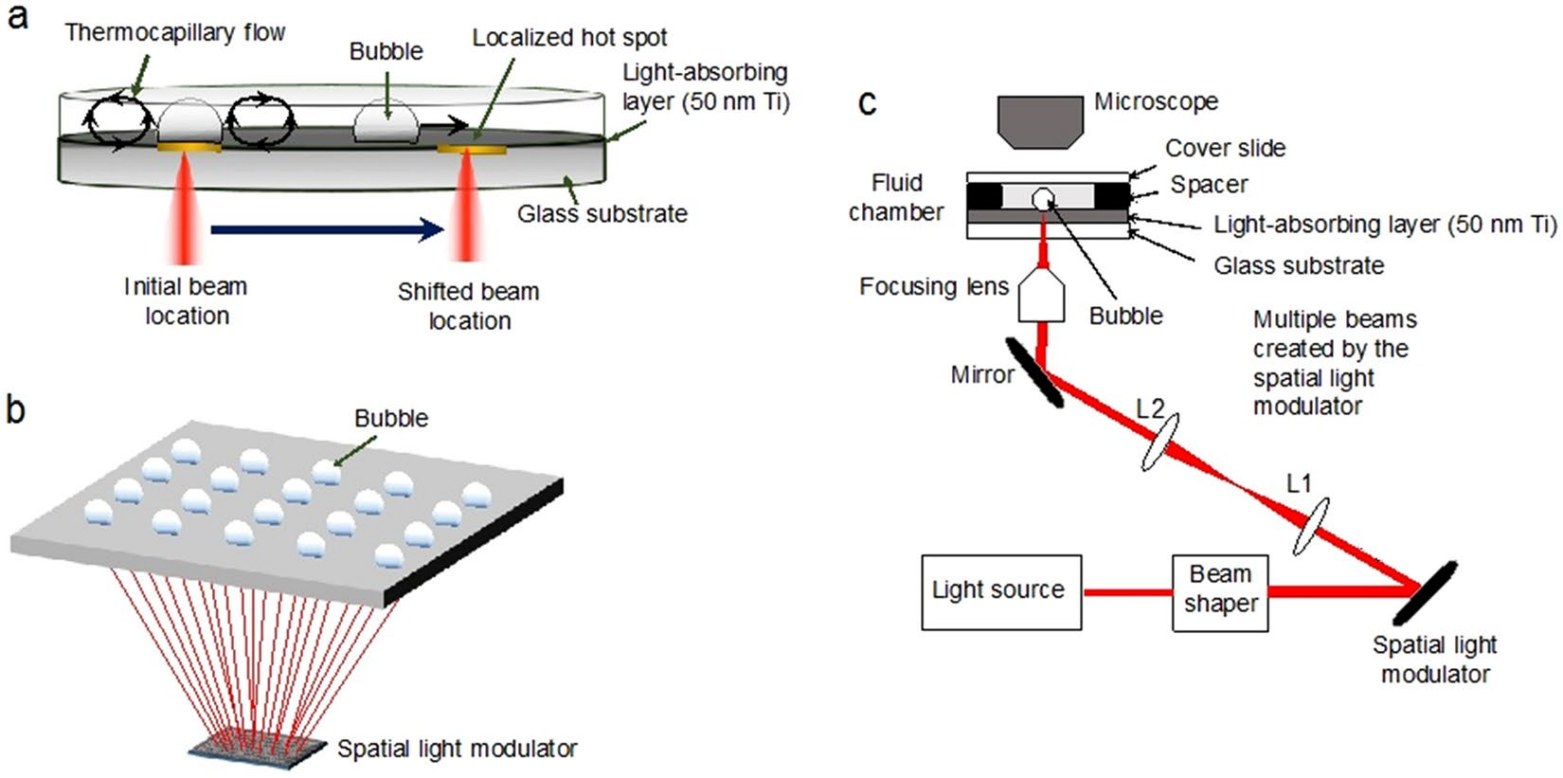
Opto-thermocapillary flow-addressed bubble (OFB) microrobots. (**a**) The OFB microrobots are gas microbubbles that are generated by the laser heating of a light-absorbing layer on the substrate. Thermocapillary forces created by the laser-generated temperature gradient are used to actuate the bubble microrobots. (**b**) A simplified schematic showing that a single laser beam can be shaped using a spatial light modulator, creating dynamic optical patterns that enable the simultaneous control of multiple OFB microrobots. (**c**) Experimental setup for the OFB microrobot system.

OFB microrobots are gas bubbles in a liquid media, and are generated by the laser heating of the floor of a fluidic chamber (Fig. 1a). The fluidic chamber used in these experiments was formed using a 500-µm spacer between a standard glass microscope slide and a glass substrate coated with 50 nm of titanium. Silicone oil (500 mPa·s) was used as the working medium in the fluidic chamber. To generate the OFB microrobots, light from an infrared laser was focused on the titanium coating; the light absorption resulted in a localized hot spot. With sufficient optical energy, the fluid above the hot spot was vaporized, generating a bubble.

The OFB microrobots are actuated by optically induced thermocapillary effects, which have been detailed in an earlier work^22^, and are described briefly here. To actuate an OFB microrobot, a radial temperature gradient is created by the absorption of a circular laser beam that is incident on the substrate. In the presence of a bubble, the temperature gradient generates a surface-tension gradient along the air/liquid interface, and a thermocapillary fluid flow around the bubble. This results in a net movement of the bubble towards the region with the highest temperature, which corresponds to the position of the laser on the substrate. Once the bubble is centered about the laser beam, the forces on the bubble are balanced, stably trapping the OFB microrobot.

The boundary condition at the liquid/gas interface of the bubble can described by the following equations:

1 $$\eta \frac{\partial u}{\partial {\boldsymbol{n}}}={\gamma }_{T}\frac{\partial T}{\partial {\boldsymbol{t}}}$$

and

2 $${\gamma }_{T}=\,\frac{\partial \gamma }{\partial T}.$$

In Eqn. 1 and Eqn. 2 ***n*** and ***t*** are the unit vectors normal and tangential to the bubble interface, *η* is the dynamic viscosity of the liquid, *u* is the tangential component of the liquid velocity vector due to thermocapillary flow at the liquid/gas bubble interface, *T* is the temperature, and *γ*_*T*_ is the surface tension^34, 35^. Eqn. 1 and Eqn. 2 show that a high thermal gradient will create a high shear stress, resulting in a stronger thermocapillary force for actuation.

A localized hot spot on the substrate is required for generating and actuating each OFB microrobot. To actuate up to 50 microrobots, a single laser beam was split into 50 spots focused on the absorbing layer of the substrate by a programmable spatial light modulator (SLM), creating the necessary hot spots (Fig. 1b,c). OFB microrobots can be generated on demand within the fluidic chamber, making it simple to create and control multiple microrobots. An OFB microrobot can be nucleated by momentarily increasing the optical power of a single laser focal point to a level sufficient to vaporize a small volume of the liquid medium. The power of the nucleating laser beam was reduced as soon as the bubble was created, to avoid an increase in the bubble size. The laser intensity used for bubble nucleation was five to six times higher than the intensity used for actuation, and is quantified in the Results section.

There are two primary considerations for nucleating and actuating OFB microrobots. The first is sufficient optical intensity; nucleation requires higher intensity than actuation. Strong light absorbance by the substrate helps to convert the incident optical energy into thermal energy. Another consideration is the degree of localization of the optical and thermal energy, which depends upon the optical elements in the experimental setup and the thermal conductivity of the absorbing material. Generally, a higher intensity of the optical beam and a lower thermal conductivity of the light-absorbing material is desirable for creating microscale hot spots.

### Microrobot control using computer-generated holograms

Using light to control the microrobot actuation force enables on-the-fly configurability of multiple OFB microrobots. This is made possible by using a SLM to display holograms that shape a single laser beam into complex optical patterns used to independently address each microrobot. The SLM was controlled using a modified version of Red Tweezers, an open-source software for the control of holographic optical tweezers created with LabVIEW (National Instruments) that renders holograms using the OpenGL Shading language^36^. Red Tweezers was modified for the requirements of OFB microrobot actuation; a bubble-collision avoidance function was added^37^, as the OFB microrobots can be susceptible to merging if they contact each other (see Supplementary Information). Another feature added to Red Tweezers was a sequence generator, a function used for creating sequences of holograms to control the motions of individual microrobots. This feature helped to enable the independent control of many microrobots in parallel.

**Figure 2:**
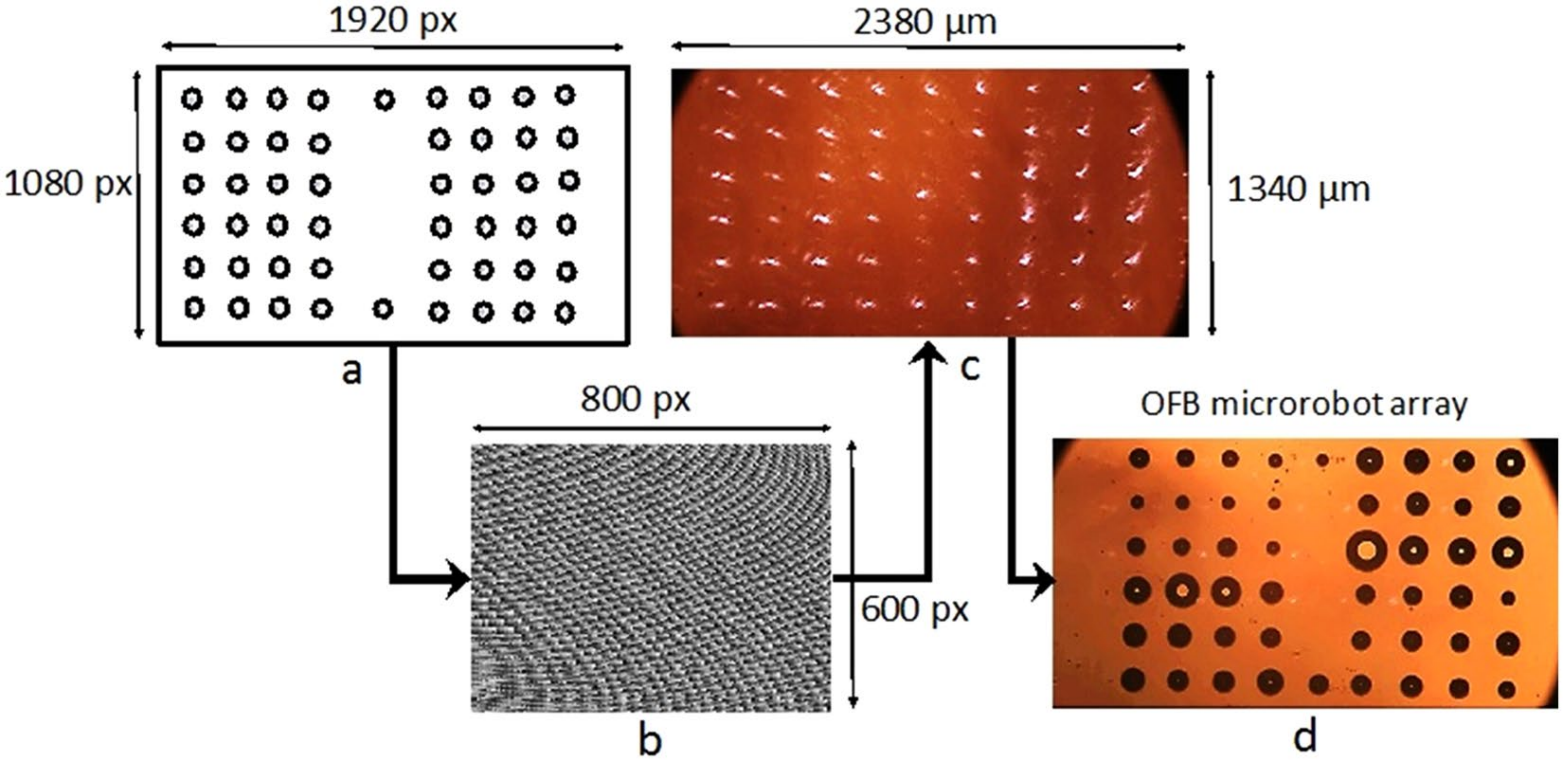
User-defined OFB microrobot control pattern generation. (**a**) A pattern of 50 focused laser spots for the control of 50 OFB microrobots created by the user. (**b**) The corresponding hologram used to create the optical pattern from a single input laser is generated and displayed on the spatial light modulator. (**c**) The optical pattern as viewed through the microscope. (**d**) Fifty OFB microrobots controlled using the optical pattern.

Control of the optical pattern enables control of the OFB microrobots. A focused point of light on the substrate is represented by a circular spot on the user interface of the control software (Fig. 2a). The coordinates of the laser focal points are sent to the OpenGL Shader hologram engine, which calculates the corresponding hologram using the direct superposition algorithm, and displays the hologram on the SLM (Fig. 2b). This computer-generated hologram controls the phase of the incident wavefront, creating the light pattern defined in the user interface (Fig. 2c). The reflected phase-modulated wavefront from the SLM was imaged on the substrate using 4-*f* imaging. The optical spot visible at the center of Fig. 2c is the zero-order spot, which was not defined in the user interface, and is an undesirable effect of using the SLM to create the optical pattern. The intensity of the zero-order beam spot is greater when the laser is split into only few beams. Thus, for the case presented here, where the laser beam is split into 50 spots, the effect of the zero-order spot on microrobot actuation was insignificant.

## Results

### Generation and independent actuation of 50 microrobots

Previously 24 OFB microrobots were independently actuated^24^, but the number of microrobots was limited by the available laser power and its conversion to a thermal gradient. The degree of parallelization in a multiple OFB microrobot system is proportional to the conversion of optical power to localized hot spots on the substrate, which is dependent on the laser intensity at the substrate and the efficiency of the conversion of light into heat. Increasing the laser intensity can compensate for losses in the optical elements or low optical absorption by the substrate, but this will increase the cost of the laser. Thus, for the system described here, the laser output power was regarded as a fixed quantity. Instead, to increase the number of OFB microrobots that could be actuated in parallel, the substrate material was changed: a titanium (Ti)-coated substrate^38^ replaced the amorphous-silicon (α-Si)-coated substrate used in previous work^22^.

**Figure 3:**
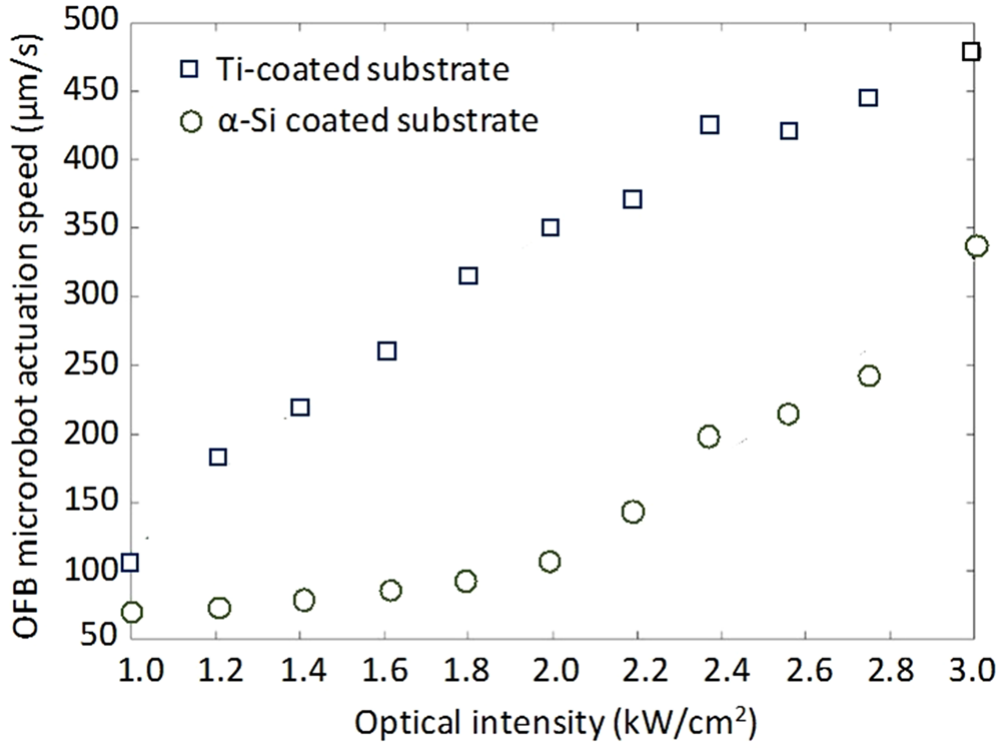
OFB microrobot actuation velocity on different substrates. The maximum OFB microrobot actuation speed for various intensities of the actuating laser beam is higher on substrates coated with 50 nm of titanium (Ti) as compared to substrates coated with 1 µm of amorphous silicon (α-Si). The size of OFB microrobots was kept constant during these measurements.

The absorbing substrate used in previous microrobot experiments consisted of a 1-mm-thick glass slide coated with 100-nm-thick layer of indium tin oxide (ITO) and 1-μm-thick layer of α-Si^24^. The Ti-coated substrate absorbs 16% more light than the α-Si-coated substrate, as measured using an optical power meter (Newport 1830C). This translated into a higher OFB microrobot actuation velocity compared to an α-Si-coated substrate (Fig. 3). However, optical intensities above 2 kW·cm^−2^ resulted in growth of the bubble size, which is not desirable during OFB microrobot actuation (Supplementary Information, Fig. S1). To avoid unwanted bubble growth, the optical intensity of this system was kept near 1.35 kW·cm^−2^ during actuation, although the bubbles still increased in size at an average rate of 0.5 µm·min^−1^ due to the continuous laser illumination. The microrobot size depends on the laser intensity and the duration of illumination^39^ (see Supplementary Information). Reducing the size of an OFB microrobot can be achieved by setting the actuating optical intensity to zero (Supplementary Information, Fig. S2) which can be utilized to control the bubble size.

At an optical intensity of 1.35 kW·cm^−2^, the Ti-coated substrate results in approximately 25% faster actuation speeds compared to the α-Si-coated substrate. Previously, 24 OFB microrobots were generated on an α-Si-coated substrate with a total laser power of 195 mW, resulting in 8.1 mW for each optical spot. In this work, 50 OFB microrobots were generated on a Ti-coated substrate, with a laser power of 330 mW, resulting in 6.6 mW for each optical spot. The minimum power required for bubble actuation on the α-Si-coated substrate was 23% higher than the power needed to actuate the same size bubble on a Ti-coated substrate. This suggests that 23% more OFB microrobots can be generated on a Ti-coated substrate.

In addition to the amount of light absorbed by the substrate, the actuation speed is also affected by the thermal gradient of the localized hot spot. A higher thermal gradient results in stronger thermocapillary effects and faster OFB microrobot actuation. The thermal gradient on Ti- and α-Si-coated glass substrates was measured by filling the fluid chamber with 1% poly(*N*-isopropyl acrylamide (PNIPAAm) in phosphate-buffered saline (PBS) solution. The PNIPAAm becomes insoluble in water when the temperature exceeds 32 °C ref. ^40^, creating a noticeable gel under optical microscopy^5^. The laser beam was focused on the absorbing layer of the fluid chamber, in the same setup shown in Fig. 1c, and the laser power was gradually increased until an OFB microrobot was nucleated in the 1% PNIPAAm solution. When the bubble is nucleated, the temperature of the liquid should be 100 °C. There was an observable gelled area of PNIPAAm around the nucleated bubble, with a boundary representing the phase-transition temperature of the PNIPAAm. Thus, the temperature gradient can be measured by assuming the temperature at the center of the laser focus is 100 °C and the temperature at the edge of the area of gelled PNIPAAm is 32 °C. Using this method, the temperature gradient was measured to be 1.08 °C·µm^−1^ on the Ti-coated substrate and 0.67 °C·µm^−1^ on the α-Si-coated substrate. The approximately 60% higher thermal gradient on the Ti-coated substrate, along with the increased absorption of the laser light, contributes to higher actuation speeds observed on these substrates (Fig. 3).

**Figure 4:**
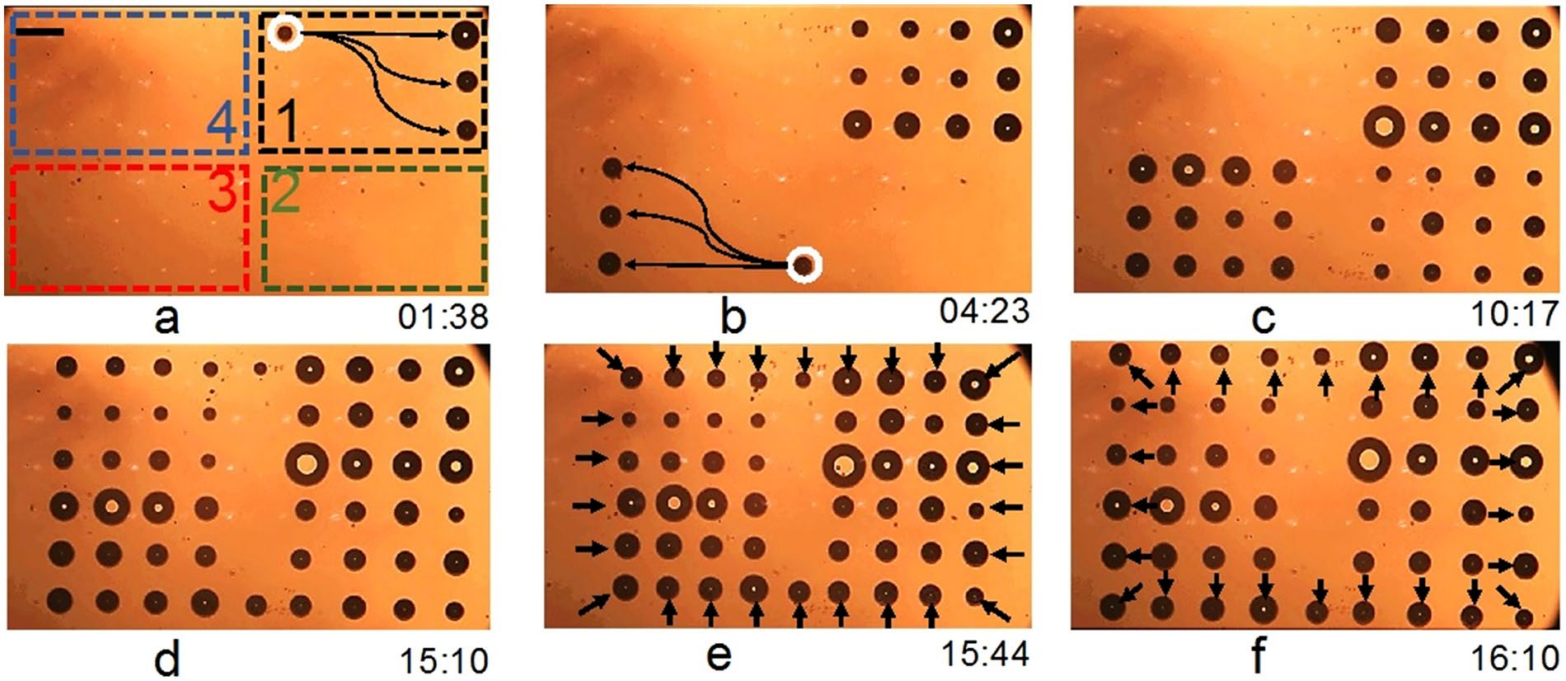
Generation and independent actuation of 50 OFB microrobots in parallel. (**a**) Top view of the workspace, virtually divided into four quadrants as labeled. The OFB microrobots were serially generated by the laser spot marked with the white circle. After generation, the bubbles were moved along trajectories indicated by the arrows using additional actuation laser spots. (**b**) Twelve OFB microrobots were generated in quadrant 1. Each microrobot is held in place by its own dedicated actuation laser spot. The laser beam marked with the white circle nucleated the microrobots at quadrant 3, following the same procedure used for the microrobots in quadrant 1. (**c**) and (**d**) Fifty microrobots were serially nucleated by the laser beams marked with the white circles in (**a**) and (**b**). (**d**) As the serial nucleation occurred in quadrants 1, 3, 2, and 4, the microrobots were assembled in a rectangular matrix. (**e**) The array of 50 microrobots was made to contract towards the center of the workspace (arrows indicate the approximate direction of motion during this operation). To complete the contraction operation, each microrobot needed to move in a different direction, thus demonstrating the independent actuation of 50 OFB microrobots in parallel. (**f**) The array of microrobots was expanded outwards, demonstrating the parallel, independent actuation of each microrobot in a different direction (arrows indicate the approximate direction of motion). Scale bar: 150 µm. Time format: minutes:seconds.

The increased OFB actuation velocities on the Ti-coated glass substrates indicated a more efficient conversion of optical energy into thermocapillary force as compared to the α-Si-coated substrates. Thus, the Ti-coated substrates were used to increase the number of OFB microrobots that could be actuated at once. Using the experimental setup described earlier, 50 OFB microrobots, with an average radius of 35 µm, were generated in a workspace spanning 2400 µm by 1350 µm (Fig. 4). The total amount of laser power available on the substrate was 330 mW, distributed among the 50 laser spots used to actuate the microrobots. In this setup, each laser spot needs an intensity of approximately 1.35 kW·cm^−2^ to stably actuate an OFB microrobot. Although this level of optical intensity is sufficient to actuate an OFB microrobot, it is not enough to generate a microbubble. To enable the generation of the 50 OFB microrobots with the limited laser power available, the workspace was divided into four quadrants (Fig. 4a). The OFB microrobots in quadrant 1 were generated by the laser spot marked with a white circle in Fig. 4a. The optical intensity of the generating laser spot was temporarily increased to approximately 7.5 kW·cm^−2^. The bubble microrobot was generated in 2 seconds, and then the beam power was reduced to the actuation intensity of 1.35 kW·cm^−2^. The bubble generation process occurred serially for the 12 OFB microrobots in quadrant 1; after each bubble was generated, an actuation light spot was used to move the OFB microrobot into position (Fig. 4a and b). Next, the 12 OFB microrobots in quadrant 3 were generated in a similar fashion by the optical spot marked with a white circle in Fig. 4b. Subsequently, OFB microrobots in quadrant 2, then quadrant 4 were generated, followed by two more microrobots (Fig. 4c and d). The total time for generating 50 OFB microrobots was 15 minutes, 10 seconds; however, bubble generation could be done in parallel, reducing the duration of the operation, by using a laser with a higher output power. A movie showing the generation of OFB microrobots is provided in the Supplementary Information, Movie M1.

Following the generation of the 50 OFB microrobots, the parallel, independent actuation of the microrobots was demonstrated (Fig. 4e and f). The formation of the microrobots was contracted and expanded, allowing each of the 50 microrobots to be actuated in different directions at the same time. Initially, the microrobots were spaced in a rectangular pattern of 1806 µm by 1125 µm (Fig. 4d). The pattern was contracted to a rectangular arrangement of 1471 µm by 893 µm, at an average actuation velocity of 5 µm·s^−1^ (Fig. 4e). The 50 OFB microrobots were then actuated to expand the formation into a rectangular arrangement of 1786 µm by 1146 µm, at an average velocity of 7 µm·s^−1^ (Fig. 4f). During this operation, the OFB microrobots were manually controlled. The actuation speed during contraction was maintained slightly lower than the actuation speed during expansion to minimize the risk of bubbles merging. A movie showing the independent actuation of microrobots is provided in the Supplementary Information, Movie M2.

### Independent actuation of pairs and groups of microrobots in various trajectories

The OFB microrobots were also actuated in subgroups. Two subgroups of 24 microrobots were formed, and the remaining two of the 50 total OFB microrobots were actuated in linear and circular trajectories, demonstrating both group motion and individual actuation of single microrobots along distinct paths.

**Figure 5:**
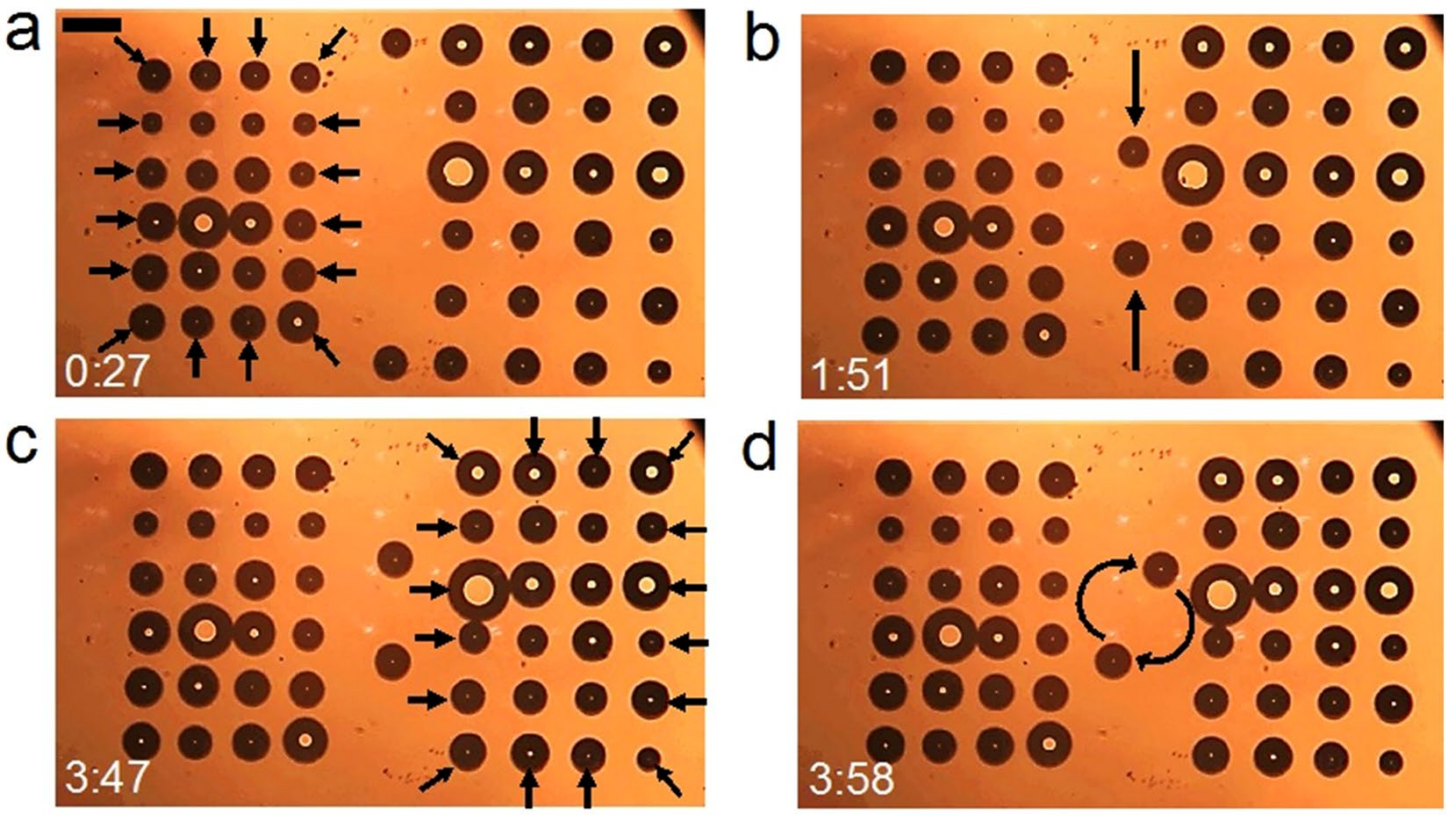
Actuation of subgroups of microrobots in various trajectories. (**a**) The matrix of 24 OFB microrobots at the left side of the workspace was made to contract, as indicated by the arrows. The other 26 microrobots were kept stationary. (**b**) A pair of microrobots were actuated towards each other while the other 48 microrobots were kept stationary, showing independent movement of a small subset of the microrobots. (**c**) The matrix of 24 OFB microrobots at the right side of the workspace was made to contract, as indicated by the arrows, while other twenty-six microrobots were kept stationary. (**d**) A pair of microrobots were actuated along a circular trajectory, first in the clockwise direction, then in the counter-clockwise direction.

First, the 24 OFB microrobots on the left side of the workspace were made to contract their formation, at a speed of 5.5 µm·s^−1^ (Fig. 5a). The other 26 microrobots remained in their respective positions, thus demonstrating actuation of a subgroup of the total available OFB microrobots. Then, the two OFB microrobots at the middle of the workspace were moved towards each other, demonstrating actuation of a smaller subgroup in a linear trajectory (Fig. 5b). The 24 OFB microrobots on the right-hand side of the workspace were then moved towards each other in parallel, while other microrobots were kept at their respective positions (Fig. 5c). The two OFB microrobots at the center of the workspace were also rotated clockwise and counter-clockwise, demonstrating the actuation of OFB microrobots along a circular trajectory with a diameter of 314 µm, at an angular velocity of 0.14 rad·s^−1^ (Fig. 5d and e). A movie showing the independent actuation of these subgroups of microrobots is included in the Supplementary Information, Movie M3.

**Figure 6:**
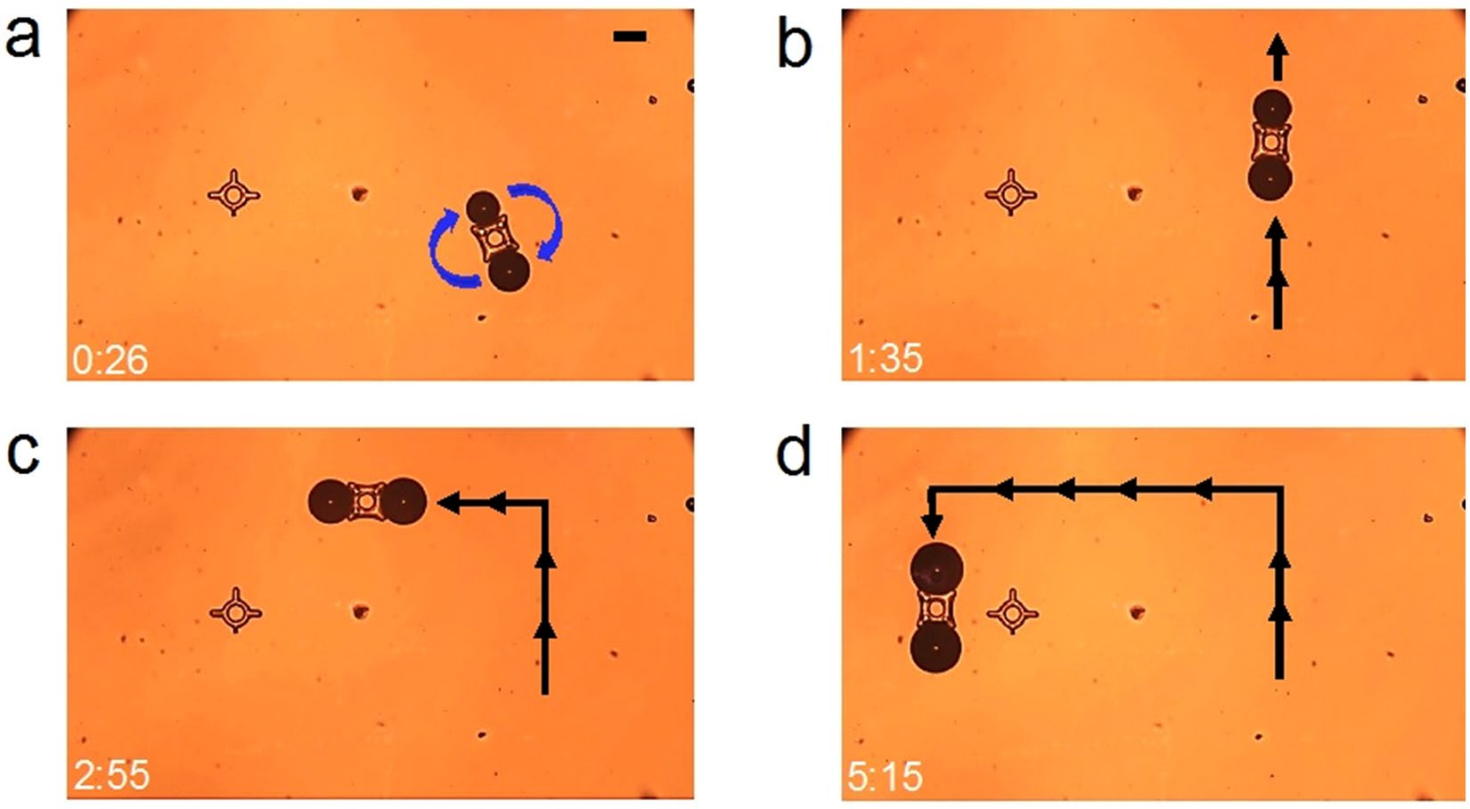
Micromanipulation of a microstructure using a pair of OFB microrobots. (**a**) A pair of OFB microrobots approached an SU-8 structure, grasped it, and rotated it towards the direction of the navigation. (**b–d**) The micro-object was transported along a piecewise linear path. The micro-object was transported along the 2017-µm-long path at the average speed of 6.0 µm·s^−1^. Scale bar: 100 µm. Time format: minutes:seconds.

### Micromanipulation of a microstructure by a pair of OFB microrobots

The independent actuation of multiple OFB microrobots enables the flexible manipulation of micro-objects, with control over orientation. This was performed using a pair of OFB microrobots on a 50-μm-high SU-8 microstructure that measured 124 μm per side, and had walls 33 μm thick that surrounded a circular hollow core (Fig. 6). A pair of microrobots were used to hold the microstructure in a pincers-like grasp, rotate the structure, and move it along linear trajectories. Upon completion of the micro-transportation, the microstructure was released at the destination location (Supplementary Information, Movie M4). Importantly, the friction and drag forces on this SU-8 structure were too large for a single OFB microrobot to overcome. Thus, the manipulation of this object was possible only because the OFB microrobots can perform cooperative micromanipulation.

### Micromanipulation by multiple OFB microrobots

**Figure 7:**
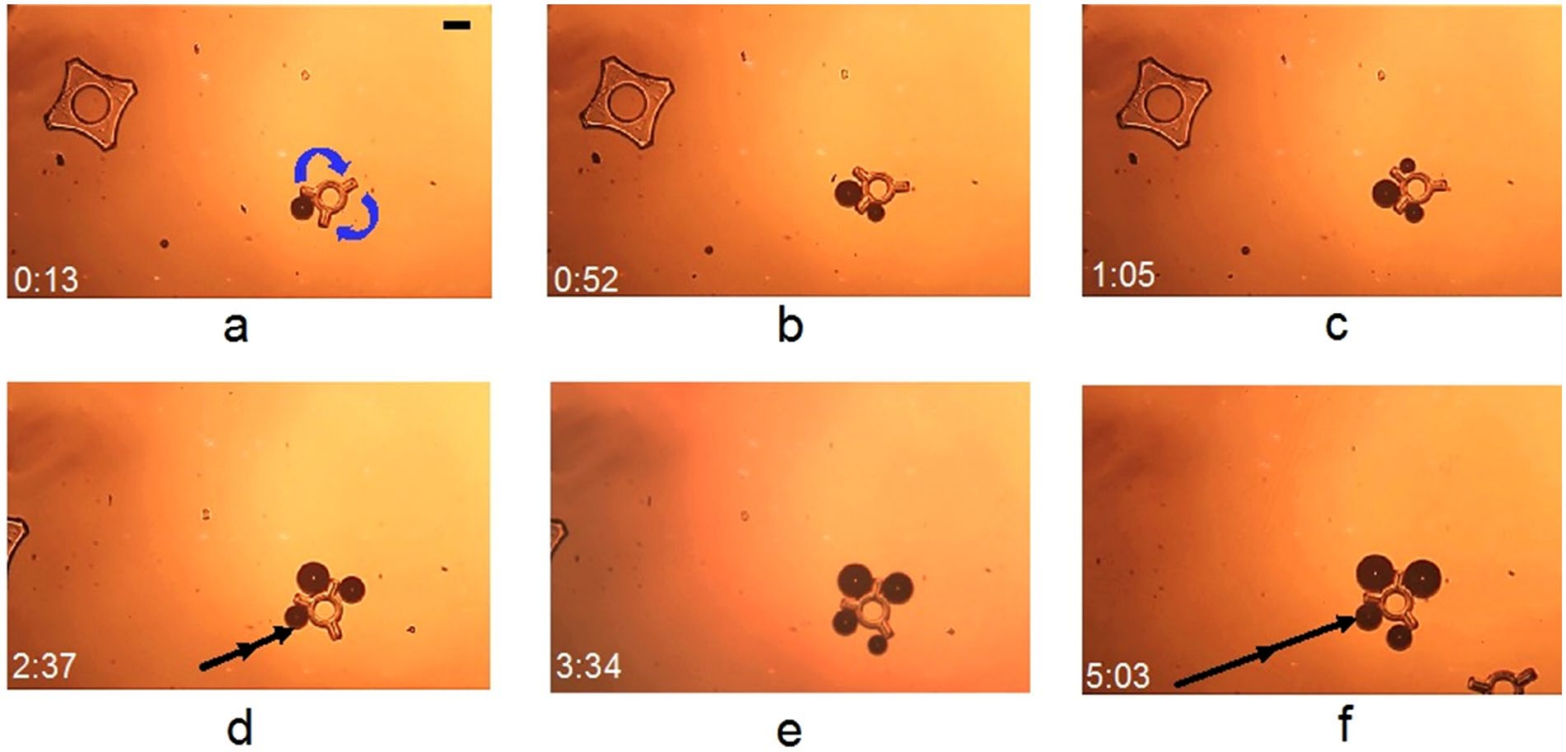
Micromanipulation by multiple microrobots. (**a**) A single OFB microrobot attempted to transport a star-like SU-8 microstructure but could only effect limited rotational movement. (**b**) A failed attempt to move the microstructure by two OFB microrobots. (**c, d**) Three OFB microrobots were able to transport the microstructure along a linear path at a speed of 8 µm·s^−1^. The micro-object was transported over a distance of 490 µm. (**e,f**) Four OFB microrobots grasping the same microstructure and transporting it along the similar linear trajectory. The micro object was carried over a distance of 1890 µm at 22 µm·s^−1^. Scale bar: 100 µm. Time format: minutes:seconds.

The independent operation of OFB microrobots allows them to perform micromanipulation tasks that may not be achievable by a single microrobot. For example, a star-like SU-8 microstructure was transported using multiple OFB microrobots (Fig. 7). The microstructure has three arms, each with a length of 100 µm, radiating from a hollow circular core with a diameter of 100 µm. The friction and drag forces on this microstructure make it difficult for one OFB microrobot to move. A single microrobot was able to rotate the structure (Fig. 7a), but could not translate it. Even the cumulative effort of two OFB microrobots did not generate sufficient force to move the microstructure (Fig. 7b). However, the combined force of three OFB microrobots was sufficient to move the structure in a linear trajectory at 8 µm·s^−1^ (Fig. 7d). The manipulation speed was increased to 22 µm·s^−1^ along the same straight path when four OFB microrobots were used to move the object. A movie illustrating the micromanipulation is provided in the Supplementary Information, Movie M5.

To quantify the effect of moving an object using varying numbers of OFB microrobots, one to four OFB microrobots were used to transport a 500-µm-diameter glass bead. A single microrobot moved the bead at a maximum velocity of 61 µm·s^−1^ (443 nN), and two microrobots working together moved the same bead at 92 µm·s^−1^ (669 nN). (The forces were calculated from the maximum velocity using Stokes’ Law with Faxen’s correction^41^). The speed of manipulation increases as more microrobots are used, although this effect begins to saturate for more than three microrobots (Supplementary Information, Fig. S4). Part of the reason for the saturation is that the forces exerted by four or more microrobots were not entirely in the direction of motion of the bead, so adding additional microrobots did not result in a linear increase in the transportation velocity (Supplementary Information, Fig. S5). Thus, the optimum number of microrobots required for manipulation of a microstructure partly depends on the shape and size of the microstructure.

### Cooperative microrobot transportation of multiple micro-objects

**Figure 8:**
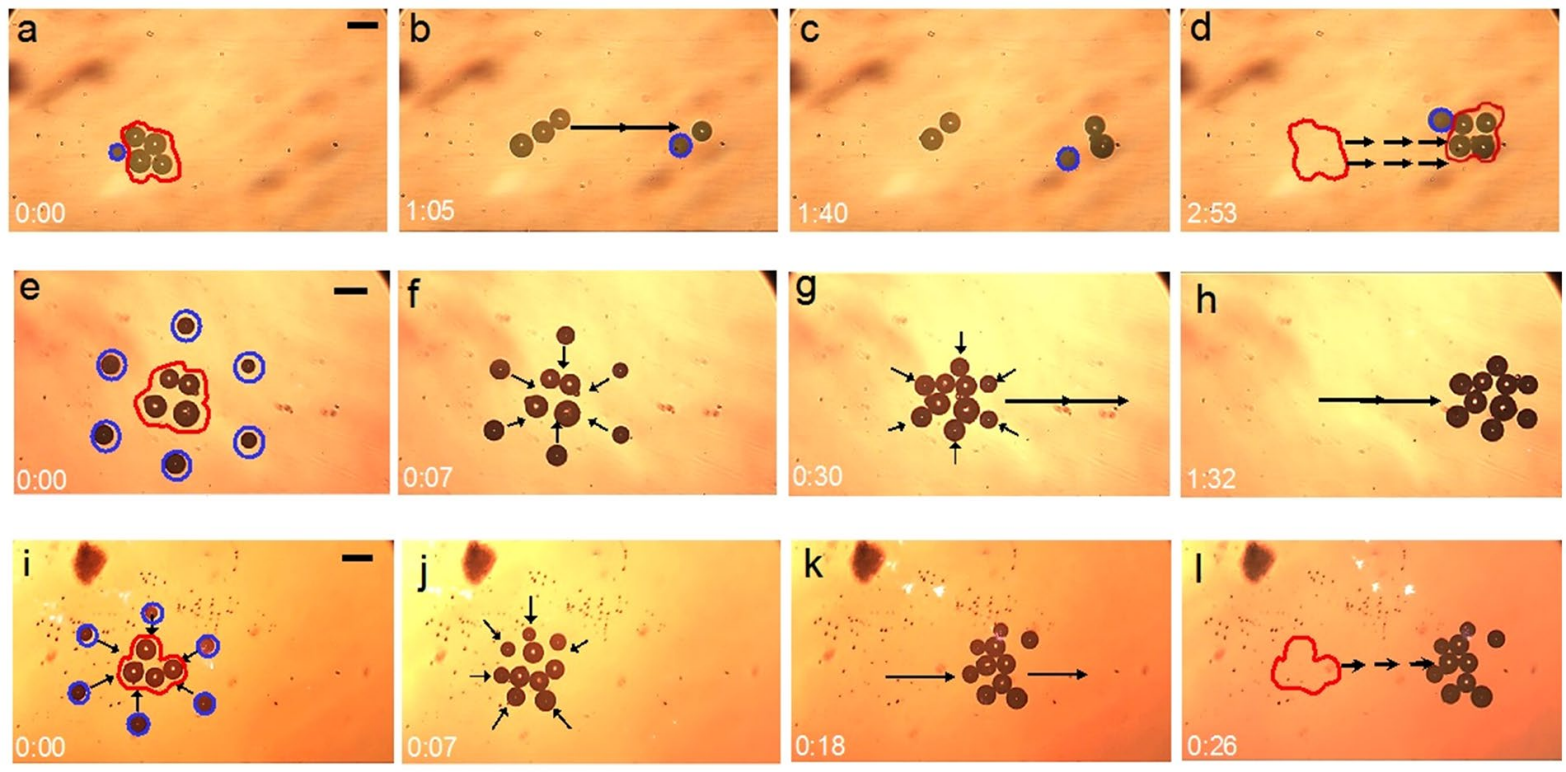
Cooperative microrobot transportation of multiple micro-objects. (**a–d**) An assembly of four glass beads was transported serially using a single OFB microrobot. (**a**) The OFB microrobot is circled in blue, and the four glass beads are enclosed by a red line. (**b–d**) The four glass beads were serially transported 844 µm away from the initial location, over an assembly time of 2 minutes and 53 seconds. (**e–h**) Manually controlled cooperative microrobot transportation of multiple micro-objects. (**e**) Six microrobots are positioned equidistant from the glass beads. The microrobots are circled in blue, and the glass beads are enclosed by a red line. (**f**) The OFB microrobots were manually controlled to approach the beads to grasp them. (**g,h**) The glass beads were grasped by the microrobots, and the beads were transported to the delivery location. The assembly time was 1 minute, 32 seconds. (**i–l**) Automated cooperative microrobot transportation of multiple micro-objects. (**I,j**) The microrobots, circled in blue, grasped the glass beads enclosed by a red line. (**k**) The glass beads were grasped by the OFB microrobots and transported to the desired location. (**l**) The glass beads were transported from the initial position to the final destination in 26 seconds.

The potential for the rapid delivery of multiple payloads is demonstrated by using multiple OFB microrobots to perform the simultaneous transportation of multiple micro-objects, as compared to a single microrobot performing the same task (Fig. 8). The micromanipulation time using a single microrobot, six microrobots controlled manually, and six microrobots controlled automatically were consecutively 2 minutes 53 seconds, 1 minute 32 seconds, and 26 seconds.

The baseline experiment used a single OFB microrobot to transport the 64-µm-radii (average) glass beads from their initial locations to a desired location (Fig. 8a–d). Since there was only a single microrobot, this was a serial operation. The center of the assembly location was 844 µm away from the initial positions of the beads (Fig. 8a–d), so the microrobot has to travel a total distance of more than 5908 µm during the assembly operation. The average transportation velocity was 34 µm·s^−1^, resulting in an assembly time of 2 minutes, 53 seconds (Supplementary Information, Movie M6).

In contrast, cooperative manipulation using multiple microrobots accomplished the transportation of the glass beads in 1 minute, 32 seconds (Fig. 8e–h). A different set of four glass beads with an average radius of 64 µm was transported the same distance as before, but this time all the beads were transported at once. In this case, six microrobots were moved by the user into a configuration that surrounded the beads (Fig. 8e–f), then the microrobot group was used to transport the all the beads to the desired location (Fig. 8g,h; Supplementary Information, Movie M7). The average speed of manipulation was 13.6 µm·s^−1^, which is 60% less than in the single-microrobot-assisted manipulation. However, despite the lower manipulation speed, there was a 50% reduction in manipulation time because all the beads were transported at once. The lower manipulation speed during the transportation of multiple micro-objects was partially to reduce the risk of bubbles merging. The adjacent microrobots were spaced slightly less than the diameter of the beads under manipulation to securely cage the beads, but the close proximity of adjacent microrobots meant that there was a risk of OFB microrobots merging while carrying the payload. Thus, during the manual operation of transporting multiple micro-objects simultaneously the actuation speed was varied from 11 µm to 16 µm, which allowed the operator to maintain a stable trapping formation of the microrobots.

Simultaneous transportation of the four glass beads was also carried out by controlling the OFB microrobots using an automated sequence (Fig. 8i–l). In the automated sequence, six OFB microrobots were used to grasp an assembly of four glass beads of 62-µm-radii (average) and transported them the same distance as in previous experiments, but by controlling the microrobots without continuous human interaction. The total operation of approaching the beads, grasping, and carrying the payload by six microrobots was initiated with a single click of a button on the user interface. The automated simultaneous transportation of multiple micro-objects was accomplished by running 34 sequences of frames of the optical patterns at an interval of 750 ms per frame. This controlled the formation of the microrobots that transported the payload 835 µm from the initial position in 26 seconds, at an average transportation speed of 46 µm·s^−1^ (Supplementary Information, Movie M8).

Automatic cooperative microrobot transportation of the microbeads improved the assembly further: it resulted in an 80% reduction in assembly time compared to single-microrobot manipulation, and a 66% reduction in assembly time compared to manually controlled multiple microrobot transportation. In both the single-microrobot micromanipulation and the manual multiple microrobot transportation, the operator controls the microrobots using a computer mouse. Using the control interface, a human operator can control only one microrobot at a time during the payload transportation. Using the automated control, each microrobot can be independently actuated at the same time to adjust the trapping formation for efficient delivery of the beads, resulting in a shorter assembly time. For example, in this experiment, the microrobots were concentrated on the trailing edge of the cluster of beads, so that more microrobots were engaged in the transportation of the beads. Automatic actuation also allows the independent control of multiple OFB microrobots along the different directions at different velocities at the same time, demonstrating actuation that is uncoupled to each other (Supplementary Information, Fig. S3).

## Discussion

Light was used to control the actuation of OFB microrobots, providing flexibility and on-the-fly adaptability for various micromanipulation tasks. Furthermore, optical control makes it straightforward to independently control many microrobots at once. This was demonstrated with by the independent actuation of 50 OFB microrobots in parallel. The microrobots can work cooperatively, as shown by the micromanipulation of micro-objects using a pair of microrobots, the transportation of a micro-structure using multiple microrobots, and the simultaneous transportation of multiple micro-objects by a team of microrobots. Independent actuation of multiple microrobots resulted in increased forces exerted on objects under manipulation, and shorter completion times for tasks like the transportation of multiple micro-objects. Further automation of the control system, as well as the implementation of motion planning and formation control algorithms, will further enhance the microassembly capabilities of the OFB microrobot system.

The current OFB microrobot system is limited to the micromanipulation of objects in 2D, as the bubbles are formed and actuated on the surface of the substrate. Most microrobotic systems have the same limitation, with the exception of some recently demonstrated 3D microassembly platforms^42^. However, it may be possible to implement layer-by-layer 3D microassembly in the OFB microrobot system, in a manner similar to methods used in other systems that generate forces near a surface^43^.

The liquid media used in the experiments presented here was silicone oil, instead of cell culture media or buffer solutions such as PBS. However, the mechanism of OFB microrobot actuation is compatible with aqueous solutions, including PBS^3, 5^. Silicone oil was used in these experiments due to its lower thermal conductivity compared to water. This helps to maintain the thermal gradients necessary for the actuation of many OFB microrobots, which is limited by the output power of the laser that was available. Actuation of 50 OFB microrobots is feasible in aqueous solutions if there is sufficient optical intensity at the substrate (approximately 400 kW·cm^−2^) ref. ^5^. This can be accomplished by using a higher-power laser, and reducing power losses in the optical system (the lenses used here are designed for visible wavelength transmission, and have increased losses for infrared light). Even without actuation in water, OFB microrobots actuated in silicone oil can manipulate cells seeded in hydrogels^44^ or water droplets^45^, as silicone oil is biocompatible^46^ and oxygen permeable^47^. Microrobotic manipulation in silicone oil can also be used to transport droplets of immiscible fluid for clinical diagnostics^48–50^ and to manipulate droplets with encapsulated biological materials^49^.

## Methods

### Experimental Setup

Figure 1c shows the optical setup used for creating and actuating fifty OFB microrobots. The light source was a 1064-nm single-mode (TEM00) linearly polarized laser (Laser Quantum, Ventus 1064, 1.5 + W). The 3.3-mm-diameter beam from the laser was incident on the 3.3 mm-diameter input pupil of a 3X beam expander, resulting in an approximately 10-mm-diameter collimated beam. The collimated beam was incident on a spatial light modulator (SLM) from Hamamatsu (LCOS-SLM X10468–07). This SLM alters the phase of the incident beam, and has an effective area of 16 mm by 12 mm. In this experiment, the SLM was used to split a single input laser beam into the optical patterns used to actuate multiple OFB microrobots. The phase-modulated wavefront from the SLM was 4-*f* imaged (focal length *L*_*1*_ = 100 mm, focal length *L*_*2*_ = 50 mm) on the back focal plane of a long-working-distance 10x objective lens (Mitutoyo, 0.28 N.A.), which focused the light onto the absorbing layer of the substrate. The substrate was a glass microscope slide coated with 50 nm of titanium, and formed the floor of the fluidic chamber for the OFB microrobots.

### Fluidic chamber

A fluidic chamber was established on the substrate using 500-µm spacer consisting of double-sided polyimide tape between a glass microscope slide and the titanium-coated glass slide. Silicone oil (Fisher Scientific, S159–500) was used as the working medium in the fluidic chamber.

### Image recording & processing

The OFB microrobot actuation and micromanipulation video was recorded using a CMOS camera (Allied Vision Technologies, Prosilica GE 1910) at the resolution of 1920 pixels by 1080 pixels. The workspace was observed using 2.5x microscope objective lens that provided a field-of-view of 2380 µm by 1340 µm. The microrobot sizes and the micromanipulation distances were measured from the camera images. Each image pixel corresponded to a physical dimension of 1.24 µm on the substrate.

### Data availability

The data described and generated from these experiments are available from the corresponding author upon request.

## Electronic supplementary material

Supplementary Information

Movie M1

Movie M2

Movie M3

Movie M4

Movie M5

Movie M6

Movie M7

Movie M8

Movie M9
