# Supplementary material for: Cooperative Micromanipulation Using the Independent Actuation of Fifty Microrobots in Parallel
Source: Sci Rep. 2017 Jun 12;7:3278. doi: 10.1038/s41598-017-03525-y (PMC5468299; doi:10.1038/s41598-017-03525-y)
Supplement: Supplementary file 1 — Supplementary Information [file 41598_2017_3525_MOESM1_ESM.pdf]

Supplementary Information to

Cooperative Micromanipulation Using the Independent Actuation of Fifty Microrobots in Parallel

M Arifur Rahman, Julian Cheng, Zhidong Wang, Aaron T. Ohta

OFB microrobot size

Figure S1 shows the increase in the size of OFB microrobots as a function of time under continuous laser illumination. Each data point is the averaged diameter of 24 OFB microrobots per substrate over a 30-minute period of laser illumination. The microrobots were serially nucleated and actuated to locations distributed equidistantly over the entire workspace. The microrobots were kept stationary and illuminated by their corresponding actuation spots for 30 minutes. Each of the actuating spots had an optical intensity of approximately 1 kW/cm^2. These results show that OFB microrobot size increases after nucleation due to the continuous heating by the actuating laser beam. The rate of size increase was 2.27 µm per minute on the 50-nm-Ti-coated substrate, 2 µm/minute on the 100-nm-Ti-coated substrate, and 1.8-µm per minute on the 1-µm-α-Si-coated substrate. The higher bubble growth rate on the titanium-coated substrates are due to the higher absorbance of light energy at the laser operating wavelength.

Reducing the size of an OFB microrobot can be achieved by setting the actuating optical intensity to zero (Supplementary Information, Fig. S2), which can be utilized to control the bubble size. Another way to control the OFB microrobot size is to use a pulsed laser for microrobot actuation[23]. It is possible to independently control the sizes of all bubbles by adjusting the power distribution among all the light spots. However, in the current control system, this operation needs to be manually performed by the operator, and thus was not done in these experiments. A closed-loop control system that can automatically adjust the optical power to maintain the size of all the microrobots is currently under development. This work did not focus on precise control of bubble sizes or exploring the range of bubble sizes that can be manipulated. However, OFB microrobots as small as 7 µm in diameter[23] and as large as 472 µm in diameter[22] have been actuated using the same method. In this work, the microrobots had diameters ranging from 30 µm (Fig. 4d) to 216 µm (Fig. S2).

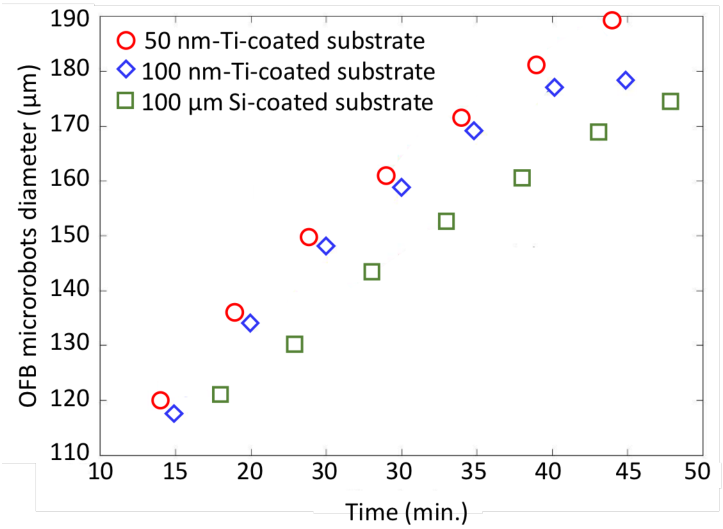

Figure S1 | OFB microrobot generation on different substrates. The average diameter of OFB microrobots over time on different substrates. The diameters are an average of a regularly spaced array of 24 microrobots over the 2380 µm by 1339 µm working area.

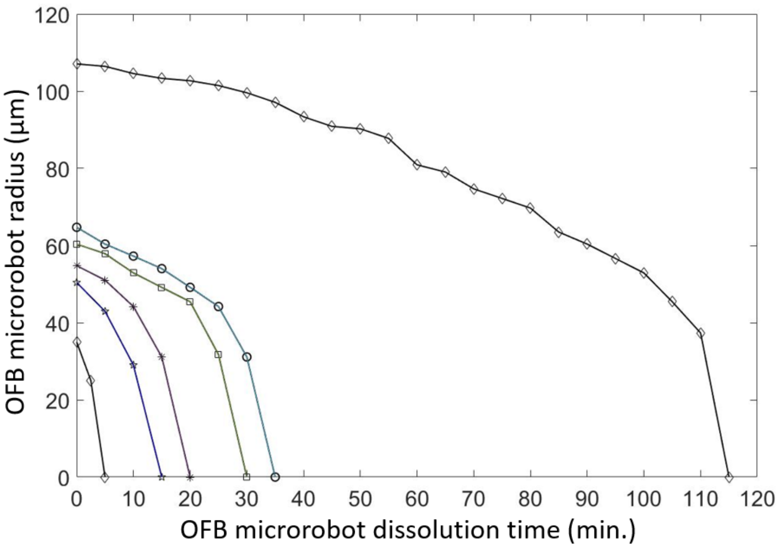

Figure S2 | Dissolution rates of OFB microrobots in silicone oil. Different sizes of OFB microrobots dissolve in silicone oil at varying rates once the actuating laser power is set to zero. Microrobots with radii of 50 μm or larger take significant time to dissolve completely. When the microrobot radius is close to 25 μm, the microrobot dissolves within four minutes. The minimum and maximum average rate of decrease in radius for 108 μm-radii and 35 μm-radii OFB microrobots is approximately 0.93 $\mu m \cdot min^{-1}$ and 7 $\mu m \cdot min^{-1}$, respectively.

The bubble size is proportional to the intensity of the laser illumination. Thus, bubble size is also dependent on the diameter of the laser illumination, if the laser power is held constant. Laser illumination with a higher intensity generates a higher thermal gradient, which is favorable for generating smaller OFB microrobots. The diameter of the optical patterns on the substrate is controlled by the hologram used to create the patterns, and the focusing objective lens (Fig. 1c). An objective lens with higher magnification will focus the optical pattern more tightly, resulting in smaller optical spots at the image plane. In this work, a 10x objective lens produced 20-μm-diameter optical spots. Using a higher-magnification objective lens will create smaller, higher intensity optical spots, resulting in higher thermal gradients. This is favorable for creating smaller OFB microrobots during nucleation, and can generate stronger thermocapillary flow around the microrobots, increasing actuation speeds. However, a higher-magnification objective lens will also proportionately reduce the working area of the microrobots, as the overall area of the optical pattern will be smaller.

Merging of OFB microrobots

As OFB microrobots are gas bubbles in a liquid medium, there is a risk of microrobots merging if the bubbles collide. Mitigating the risk of merging is an important function of the OFB microrobot control system. It was observed that two bubbles in contact do not merge, unless additional energy is provided, such as laser illumination or if the bubbles have some motion relative to each other. An example is shown in Supplementary Figure S3 (b) and (c), where two bubbles were in contact with each other during actuation, but did not merge. However, the risk of merging increases significantly if two bubbles being pushed or pulled towards each other by external forces. During bubble generation and actuation, an opto-thermocapillary force exists that is proportional to the thermal gradient of the hot spot actuating the microrobot. When two OFB microrobots are actuated with two optical spots of equal intensity, the thermocapillary flow of each microrobot cancels each other, so there is no force pulling or pushing the bubbles towards each other. If a stronger optical intensity is used to actuate one microrobot, the stronger thermocapillary flow around the this microrobot will pull the other microrobot toward it, posing a significant risk of merging the bubbles. However, empirical observations have shown that the risk of bubble merging is not significant until the optical intensity of one optical spot is three times less than an adjacent optical spot. This merging risk was present during the OFB microrobot generation, since five to six times higher optical intensity was used to generate the microrobots as compared to the optical intensity used to actuated the microrobots. Merging was prevented by creating OFB microrobots away from actuated microrobots.

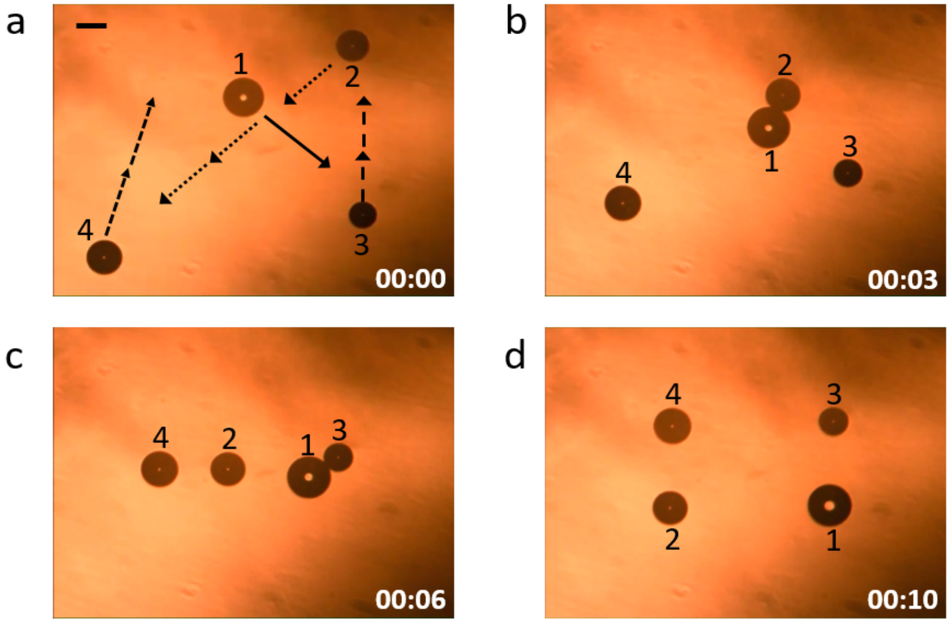

Figure S3 | Independent actuation of multiple microrobots at different velocities. (a) Four OFB microrobots were generated at random locations within the workspace. Each microrobot was actuated along a separate trajectory, as indicated by the arrows. (b) The microrobots are actuated towards their respective destinations. Microrobots 1 and 2 contacted each other, but did not merge. (c) Microrobots 1 and 3 contacted each other, but did not merge. (d) The four microrobots have reached their destination location, forming a rectangular shape. Microrobots 1 to 4 were independently actuated along different linear trajectories at velocities of 47.2, 83.1, 44.9, and 64.7 μm·sec^{-1}, respectively. Scale bar: 100 μm. Time format: minutes:seconds.

OFB microrobot velocities

The maximum OFB microrobot-assisted transportation speed demonstrated was 162 μm·s^{-1} (Supplementary Information, Fig. S4) and the minimum transportation speed demonstrated was 6 μm·s^{-1} (Fig. 6). A single OFB microrobot moving by itself (without pushing an object under manipulation) has been actuated up to 475 μm·s^{-1} (Fig. 3) under an optical intensity of 3 kW·cm^{-2}. The bubble actuation speed and micromanipulation speed can be increased by illuminating an OFB microrobot with higher optical intensity.

Micromanipulation using OFB microrobots

The size of objects that can be manipulated using this system depends on the mass, density, and shape of the object. This method has been used to transport polystyrene beads as small as 20 μm in diameter[23] and glass beads as large as 500 μm in diameter (Supplementary Information, Fig. S4). Heavier micro-objects can be manipulated by employing multiple microrobots, but the number of microrobots that can be used are limited by the cross-sectional area of the object. The shape of the micro-object also plays a significant role in manipulation. Spherical objects have minimal contact with the substrate, resulting in lower friction. Flat microstructures with more contact to the substrate experience higher friction, and thus require more force to move.

As more microrobots are used to cooperatively transport the same micro-object, the transportation velocity increases, although it saturates with increasing numbers of microrobots (Fig. S4). One, two, or three microrobots can be moved completely parallel to the direction of motion of 500-μm-diameter glass bead under manipulation, so the force exerted upon the bead is entirely towards the direction of the transportation (Fig. S5). However, as more microrobots are added, their direction of motion is not completely aligned with the direction of the bead motion (Fig. S5d). This contributes to the observed saturation in transportation velocity.

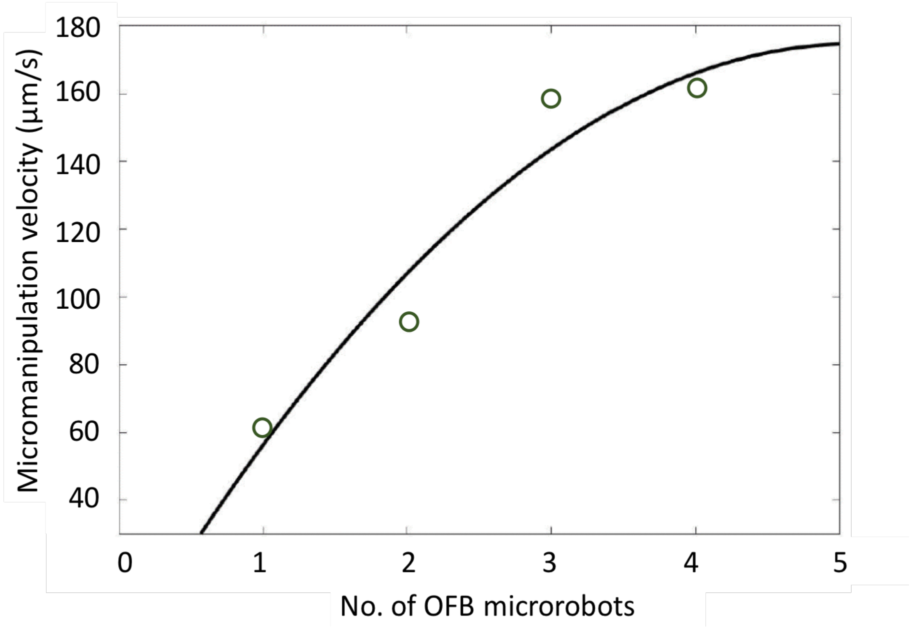

Figure S4 | Single micro-object manipulation with increasing numbers of microrobots. The maximum velocity achieved while transporting a 500-µm glass bead increases as more microrobots contribute to the manipulation, due to the increased actuation force. However, this effect saturates, as using four microrobots starts to increase the cross-sectional area of the bead-microrobot cluster, increasing drag.

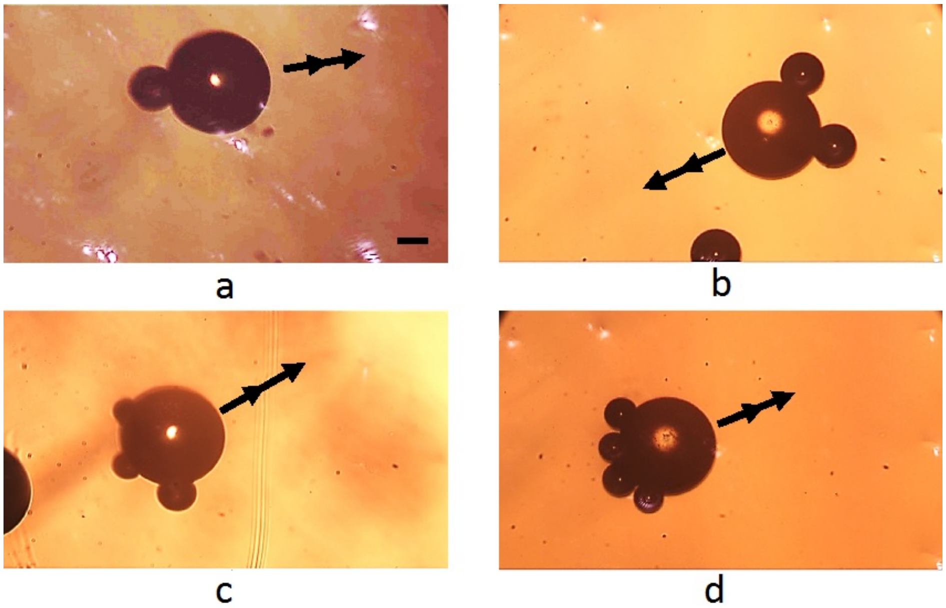

Figure S5 | Micro-object manipulation using single and multiple OFB microrobots. The images shown here correspond to the experiments in Fig. S4. (**a**) A single OFB microrobot transports a 500-µm-diameter glass bead. (**b**) Two microrobots transported the same bead. (**c**) Three microrobots transporting the bead. (**d**) Four OFB microrobots pushing the same glass bead. The arrows indicate the direction of transportation in each image. Scale bar: 100 µm.

Independent control of multiple OFB microrobots

To demonstrate that the OFB microrobots described in this paper meet this definition of "independent control," mentioned in the introduction of the main paper, additional experimental results have been added as Fig. S3. Independent control was demonstrated by actuating four OFB microrobots from random locations in the workspace to make a rectangular formation. The microrobots were actuated along different linear trajectories in parallel, and the actuation velocity of each microrobot was different (47.2, 83.1, 44.9, and 64.7 $\mu m \cdot s^{-1}$). The movie illustrating this independent actuation is provided as Supplementary Information, Movie M9.

Movie M1 | Generation of 50 OFB microrobots. Fifty opto-thermocapillary flow-addressed bubble (OFB) microrobots are serially generated and actuated to form a matrix. The playback speed of this movie is 22x real-time. The University of Hawaii at Manoa logo is used with permission from the University of Hawaii.

Movie M2 | Independent actuation of 50 OFB microrobots. A matrix of 50 OFB microrobots is made to expand and contract, demonstrating independent multi-directional actuation of each microrobot in parallel. The playback speed of this movie is 2x real-time. The University of Hawaii at Manoa logo is used with permission from the University of Hawaii.

Movie M3 | Actuation of multiple microrobots in linear and circular trajectories. Two groups of 24 OFB microrobots each are actuated in turn, followed by actuation of a pair of microrobots along a linear and circular trajectory. The playback speed of this movie is 8x real-time. The University of Hawaii at Manoa logo is used with permission from the University of Hawaii.

Movie M4 | Cooperative micromanipulation using a pair of microrobots. A single microrobot does not produce sufficient force to move an SU-8 microstructure, so two OFB microrobots co-operatively transport the object along a piecewise linear path. The playback speed of this movie is 10x real-time. The University of Hawaii at Manoa logo is used with permission from the University of Hawaii.

Movie M5 | Pick-and-place micromanipulation of planar structures. One or two OFB microrobots struggle to transporting an SU-8 microstructure, but three and four microrobots can cooperatively move the object. The playback speed of this movie is 8x real-time. The University of Hawaii at Manoa logo is used with permission from the University of Hawaii.

Movie M6 | Transport of multiple micro-objects by single microrobot. A single OFB microrobot is used to serially transport four microbeads. The playback speed of this movie is 6x real-time. The University of Hawaii at Manoa logo is used with permission from the University of Hawaii.

Movie M7 | Transport of multiple micro-objects by manually controlled microrobots. Six manually controlled OFB microrobots are used to transport an assembly of four micro-objects. The playback speed of this movie is 6x real-time. The University of Hawaii at Manoa logo is used with permission from the University of Hawaii.

Movie M8 | Transport of multiple micro-objects by automated microrobots. Six automated OFB microrobots are used to transport an assembly of four micro-objects. The playback speed of this movie is 6x real-time. The University of Hawaii at Manoa logo is used with permission from the University of Hawaii.

Movie M9 | Independent actuation of multiple microrobots with different speed. Four OFB microrobots were automatically actuated along different trajectories with different actuation speed. The University of Hawaii at Manoa logo is used with permission from the University of Hawaii.

The jerky motions of the videos are due to the frame rate of the camera and the frame-by-frame incremental movement of the microrobots. The camera used for recording the videos had a frame rate of 30 frames per second. This was not synchronized with the holographic control system of the OFB microrobots, which actuates the microrobots by changing the optical pattern frame by frame. This incremental actuation of the OFB microrobots is evident in the Supplementary Information Movies M1 to M9, as noted by the reviewer. The OFB microrobot motion can be made to be smoother by using a higher frame rate, at the cost of increased computer processing.
